# Consensus to Conquer Challenges in Influenza Immunization in Pulmonology Practice in India: Proceedings of the Congregation on Influenza Vaccination in Chronic Lung Diseases (CIRCLE) Expert Committee Meet

**DOI:** 10.7759/cureus.89176

**Published:** 2025-07-31

**Authors:** Parvaiz Ahmad Koul, Abhisheka Kumar, Aditya Parihar, Ajay Verma, Ameer KA, Anil Aggarwal, Aratrika Das, G Thilagavathy, Kamal Kumar Jain, Narendra Rawal, Naveed Nazir Shah, Prashant Kumar, RP Senthil Kumar, Samir Sahu, Sujith Varghese Abraham, Vivek Vardhan Virapaneni, Zafar Iqbal, Trayambak Dutta, Manish Mahajan, Samir Desai

**Affiliations:** 1 Pulmonary Medicine, Sher-i-Kashmir Institute of Medical Sciences, Srinagar, IND; 2 Pulmonology, Netaji Subhas Medical College and Hospital, Bihta, IND; 3 Pulmonology, Indira Gandhi Government Medical College and Hospital, Nagpur, IND; 4 Respiratory Medicine/Pulmonary Critical Care Medicine, King George's Medical University, Lucknow, IND; 5 Interventional Pulmonology, KIMSHEALTH Hospital, Trivandrum, IND; 6 Respiratory Medicine, DNS Hospitals, Indore, IND; 7 Respiratory Medicine, Narayana Health, Kolkata, IND; 8 Interventional Pulmonology, Vijaya Group of Hospitals, Chennai, IND; 9 Diabetes and Endocrinology, Arya Hospital, Guwahati, IND; 10 Pulmonology, Byramjee Jeejeebhoy Medical College, Civil Hospital, Ahmedabad, IND; 11 Respiratory Medicine, Government Medical College, Srinagar, IND; 12 Respiratory, Sleep and Critical Care, IQ City Medical College and Hospital, Durgapur, IND; 13 Interventional Pulmonology, Pulmonary Critical Care and Sleep Medicine, Annai Arul Hospital, Chennai, IND; 14 Critical Care and Pulmonology, Apollo Hospitals, Bhubaneswar, IND; 15 Critical Care and Pulmonology, Ashwini Hospital, Cuttack, IND; 16 Respiratory Medicine, KIMSHEALTH Hospital, Trivandrum, IND; 17 Interventional Pulmonology, Swasa Hospital, Hyderabad, IND; 18 Pulmonology, Sleep and Medical Intensive Care, Fortis Hospital, Mohali, IND; 19 Infectious Disease, Zydus Lifesciences, Ahmedabad, IND; 20 Medical Affairs, Zydus Lifesciences, Ahmedabad, IND

**Keywords:** asthma, copd: chronic obstructive pulmonary disease, healthcare professionals, immunization barrier, influenza vaccine, public health, seasonal influenza, trivalent vaccine

## Abstract

Influenza remains a major health concern, especially for patients with chronic respiratory diseases such as asthma and chronic obstructive pulmonary disease (COPD). Despite clear evidence of its benefits, annual influenza vaccination rates in India remain critically low, particularly among high-risk populations and healthcare professionals (HCPs). Key barriers include misinformation, lack of provider recommendation, and absence of structured vaccination programs. The year-round circulation of influenza in India supports the use of both Northern Hemisphere (NH) and Southern Hemisphere (SH) vaccines, depending on the timing. Patients should be vaccinated with the latest NH or SH strain whenever they present for immunization. Recent guidelines favor trivalent vaccines due to the absence of the B/Yamagata strain. A multi-pronged strategy involving HCP advocacy, public education, improved infrastructure, and policy support is essential to improve vaccine uptake and reduce influenza-related morbidity and mortality.

## Introduction and background

The influenza virus has endured for millennia by leveraging antigenic drift and shift, exemplifying the evolutionary mandate of continual adaptation for survival [1]. It continuously evolves and adapts, thus evading the host memory immune responses and causing influenza illness and its complications, primarily through involvement of the respiratory system [2]. Every year across the globe, there are an estimated 1 billion influenza cases, of which 3 to 5 million are severe, resulting in 290,000 to 650,000 influenza-related respiratory deaths [3-6]. Patients with chronic medical disorders, including those with chronic respiratory diseases, are at a higher risk of developing influenza-related complications [7]. India has more than 70 million patients with COPD and asthma, both associated with acute exacerbations. One out of five patients (22%) hospitalized with COPD exacerbations were infected with influenza [8]. During influenza seasons, up to one-fifth of infants presenting with wheezing and roughly a quarter of adults experiencing acute asthma exacerbations may test positive for influenza viruses. Nevertheless, the worldwide prevalence of influenza virus detection in asthma exacerbation cases remains comparatively low, averaging around 10% [9-12]. When asthmatic patients become infected with influenza, it can lead to a decline in lung function and complications such as pneumonia. While influenza vaccination is regarded as the most effective prevention strategy against influenza, less than 1% of adult patients at high risk receive the annual influenza vaccine in India [3,13]. Vaccination remains a cornerstone strategy for preventing influenza transmission and associated complications. Those at greatest risk, and therefore most likely to benefit from immunization, include individuals with chronic respiratory diseases, immunocompromised states, caregivers or household contacts of high-risk groups, adults over 50 years of age, pregnant women, children, and individuals with other underlying chronic conditions. Influenza vaccines are well-established as safe and are available in several formulations, including live attenuated, inactivated, adjuvanted, cell culture-derived, and recombinant types. Their efficacy can be enhanced when administered either alongside or in sequence with other live or inactivated vaccines. Each year, influenza vaccines are manufactured with the virus strains recommended by the WHO, based on global surveillance of influenza virus strains. The recommended viruses for inclusion in the vaccine are updated annually, based on global surveillance and the likely circulating strains. Various global practice guidelines and recommendations for adult influenza vaccination are in place for different circulating influenza virus strains; however, such guidelines are lacking in India [13,14].

## Review

What is the influenza immunization rate in India in patients with respiratory illness? What are the challenges?

A recent study involving 64,714 Indian adults aged 45 years or older revealed concerning statistics regarding vaccination rates. The uptake for influenza vaccination was a mere 1.5%, while pneumococcal disease, typhoid, and hepatitis B vaccinations stood at 0.6%, 1.9%, and 1.9%, respectively, as reported by Rizvi AA and Singh A in 2022 [15]. Since healthcare professionals (HCPs) are the key drivers of vaccination, their attitudes and practices are paramount in determining vaccination uptake. Notably, only 4.4% of HCPs reported receiving the influenza vaccine 1-3 times in the past five years [16]. The low vaccination rates persist even though 95% of participants acknowledged the potential adverse consequences of influenza [17].

The most significant challenge identified was the attitude and practices of HCPs toward influenza vaccination, with abundant misconceptions and misperceptions about vaccine safety and efficacy [18]. Despite clear recommendations from the WHO and the CDC for routine influenza vaccination of healthcare workers (HCWs), the data indicate poor uptake [19]. Even after heightened awareness following the COVID-19 pandemic, vaccination uptake remained poor when it was not considered mandatory for employment [20].

These findings underscore the urgent need for targeted interventions to improve vaccination rates among both patients at high risk for influenza complications (including those with chronic respiratory disease) and HCPs. Addressing the underlying attitudes and practices toward vaccination is crucial to enhancing public health outcomes and preventing the spread of infectious diseases.

Similarly, misconceptions about efficacy and adverse events lead to barriers among patients and parents. Complex childhood/adolescent vaccination schedules, previous perceived adverse experiences, misinformation and disinformation, along with cultural, religious, social, and personal factors, contribute to parental apprehension regarding the vaccination of their children.

What is the way forward to overcome the challenges and improve annual immunization in India? What is the percentage of patients visiting pulmonology clinics every year for influenza vaccination?

To overcome the challenges and improve annual influenza immunization among patients with Chronic Obstructive Pulmonary Disease (COPD) and asthma in India, a multifaceted approach is essential. First, enhancing awareness and education through targeted campaigns can help dispel myths and emphasize the importance of vaccination for these high-risk groups. Strengthening healthcare infrastructure to ensure vaccine accessibility, especially in remote areas, is crucial. Regular training for pulmonologists and other HCPs can help them stay updated on vaccination protocols and emphasize the importance of immunization for patients with respiratory conditions. Policy and advocacy efforts should consider mandating vaccinations for HCPs working with at-risk patients (such as immunocompromised individuals), and securing increased government support for recommending and rolling out influenza vaccination among high-risk individuals. Engaging community leaders and influencers to promote vaccination and address cultural barriers can also play a significant role. Additionally, implementing robust monitoring systems to track vaccination coverage and identify areas needing improvement is vital.

Influenza vaccination uptake in patients with COPD continues to be low in India, with negligible vaccination rates reported in Burden of Obstructive Lung Disease (BOLD) study data from three centers in India [21]. Regarding the percentage of patients with COPD and asthma visiting pulmonology clinics for influenza vaccination, a study indicated that while 82.82% of physicians offered influenza vaccines to their patients, only about 32.69% vaccinated 10-25% of their patients per month. This highlights the need for targeted interventions to increase vaccination rates among patients with respiratory conditions visiting pulmonology clinics [22].

Among adults, a strong recommendation from a physician is consistently associated with higher rates of influenza vaccination. Practical approaches, such as pre-established vaccination protocols, appointment reminders, and offering the vaccine during routine visits, can further enhance uptake. Equally important is the quality of provider-patient communication. Individuals uncertain about vaccine information, especially those mistrusting public sources, are more likely to respond positively when their physician provides reassurance and personalized guidance. For caregivers and parents, allocating time to voice concerns and receive empathetic responses may encourage acceptance. Additionally, patient-centered logistics, such as welcoming clinical environments, flexible appointment scheduling, and shorter waiting periods, have been linked to increased immunization adherence [23,24].

What are the influenza vaccination guidelines in India?

Although influenza vaccination is not currently part of the Universal Immunisation Program of the Government of India, the MoHFW recommends annual vaccination for high-risk groups. These include HCWs, pregnant women, children aged 6 months to 5 years, elderly individuals (65 years and older), and individuals with chronic medical conditions such as asthma, COPD, diabetes, and heart disease [25]. Several professional medical societies in India also endorse influenza vaccination for patients under their care. The composition of influenza vaccines is updated biannually by the WHO, in February for the Northern Hemisphere (NH, which includes India) and in September for the Southern Hemisphere (SH), to ensure alignment with the most prevalent circulating strains.

In India, both trivalent and quadrivalent influenza vaccines are available. However, in accordance with recent WHO recommendations, the quadrivalent vaccine is generally preferred as it provides broader protection, covering two influenza A strains (H1N1 and H3N2) and two influenza B strains. The trivalent vaccine may still be available in some settings, but its use is gradually being phased out in favour of quadrivalent formulations, in line with global best practices. It is recommended that influenza vaccination be administered prior to the onset of the influenza season, which in India typically coincides with the monsoon and winter months. A single annual dose is sufficient for most individuals. However, children aged 6 months to 8 years receiving the vaccine for the first time should receive two doses, spaced at least four weeks apart. These recommendations aim to minimize influenza-related morbidity and mortality, particularly in vulnerable populations [26].

Healthcare personnel working in hospitals and institutional settings, including doctors, nurses, paramedical staff, and other frontline workers at risk of exposure to the influenza virus, are strongly advised to receive annual influenza vaccination. This recommendation particularly applies to those working in emergency departments, ICUs, and isolation wards where influenza patients are managed, as well as individuals assigned to screening centers during seasonal influenza outbreaks. Additionally, healthcare providers treating high-risk groups, laboratory personnel handling suspected influenza samples, members of rapid response teams deployed for outbreak investigations, and ambulance staff involved in patient transport are all considered priority groups for vaccination.

Vaccination is also recommended for all pregnant women during the influenza season, regardless of gestational age, due to the increased risk of severe outcomes in this population. The inactivated influenza vaccine is indicated for individuals with chronic health conditions such as COPD, bronchial asthma, cardiovascular diseases, chronic liver or kidney disorders, diabetes mellitus, cancer, blood disorders, and for those with immunocompromised states, including individuals receiving immunosuppressive therapy. Children with underlying chronic diseases, including asthma, neurodevelopmental conditions such as cerebral palsy, epilepsy, and intellectual disabilities, congenital or acquired heart disease, hematologic disorders like sickle cell disease, metabolic disorders, and hepatic or renal dysfunction, are also prioritized for vaccination. Furthermore, elderly individuals aged 60 years and older, along with children between 6 months and 8 years of age, are recognized as vulnerable groups and are therefore recommended to receive the vaccine [27].

In line with guidance from the MoHFW, Government of India, seasonal influenza vaccination is advised for the SH influenza season of 2025. Following WHO recommendations, the quadrivalent influenza vaccine for this season should contain the following strains: A/Victoria/4897/2022 (H1N1)pdm09-like virus (≥15 µg hemagglutinin [HA]), A/Croatia/10136RV/2023 (H3N2)-like virus (≥15 µg HA), B/Austria/1359417/2021 (B/Victoria lineage)-like virus (≥15 µg HA), and B/Phuket/3073/2013 (B/Yamagata lineage)-like virus (≥15 µg HA). These vaccine components are produced using fertilized hen’s eggs from healthy flocks and are inactivated using beta-propiolactone. Notably, adjuvanted, cell culture-derived, and high-dose influenza vaccines are currently not available in India.

What is the efficacy of influenza vaccination in patients with COPD?

Influenza vaccination plays a crucial role in reducing respiratory illnesses and mortality among patients with COPD. A comprehensive review from the Cochrane Database of Systematic Reviews (2018), involving six studies with 2,469 participants and five studies with 4,281 older or high-risk patients, demonstrated that inactivated influenza vaccines significantly lowered the number of exacerbations in vaccinated individuals compared to those receiving a placebo [28]. This reduction in exacerbations is particularly beneficial for COPD patients. Additionally, a study conducted by Huang HH et al., involving 19,788 COPD patients, found that influenza vaccination reduced the risk of respiratory failure, highlighting its importance in geriatric COPD patients [26].

Further evidence from a study by Gershon AS et al., involving 21,748 patients older than 66 years, indicated that seasonal influenza vaccination was associated with a 22% reduction in laboratory-confirmed influenza-associated hospitalizations among older adults with COPD [27-30]. This underscores the moderate effectiveness of the vaccine in preventing severe influenza outcomes in this population. Moreover, a large-scale study published by Young-Xu Y et al., involving 1,856,970 elderly COPD patients, revealed that influenza vaccination significantly reduced the risk of death by 75% from all causes, 76% from respiratory causes, and 82% from pneumonia or influenza [28-31].

In terms of preventing laboratory-confirmed influenza, a study involving 1,761 COPD patients found that the average influenza prevention rate was 40% for current-season vaccination and 24% for vaccination in the prior season [32]. Lastly, research by Mulpuru S et al., involving 4,198 COPD patients, demonstrated a 38% reduction in influenza-related hospitalizations among vaccinated individuals [30]. These findings collectively emphasize the efficacy of influenza vaccination in reducing respiratory illnesses, hospitalizations, and mortality among patients with COPD and asthma, highlighting its critical role in managing these chronic respiratory conditions.

What is the efficacy of influenza vaccination in patients with asthma?

Influenza vaccination plays a pivotal role in protecting asthma patients from severe influenza-related complications. A study conducted by Suárez-Varela MM et al., involving 582 hospitalized patients with laboratory-confirmed influenza, demonstrated a significant protective effect of vaccination in asthmatic patients [31-34]. Further research published in the Canadian Medical Association Journal (2021), involving 1,032 asthma patients, found that the average effect of influenza vaccination was 43% for current-season vaccination and 38% for vaccination in previous seasons only, indicating substantial protection against influenza [35].

Another study spanning six influenza seasons and involving 5,910 asthma patients revealed that vaccination was associated with an overall 55.0% risk reduction in laboratory-confirmed influenza infections in asthmatics [36]. A comprehensive review encompassing 35 studies with 142,519 asthma patients reported a pooled efficacy of live vaccines in reducing influenza by 81%. The same review highlighted that influenza vaccination prevented 59% to 78% of asthma attacks leading to hospitalizations [37].

These findings underscore the efficacy of influenza vaccination in reducing the incidence of influenza infections and related complications in asthma patients, emphasizing its critical role in managing and preventing severe outcomes in this vulnerable population.

What is the ideal timing for influenza vaccination, especially when there are two different strains available, NH and SH across March-July and August-February, respectively, annually in India? What is the concept of year-long vaccination with the influenza vaccine?

Influenza viruses exhibit continuous antigenic variation, necessitating regular updates to vaccine composition. To support this, the WHO conducts biannual evaluations of influenza surveillance data collected from over 100 countries. These reviews, held in February and September, inform strain selection for the formulation of seasonal influenza vaccines tailored separately for the NH and SH [26].

Although India is geographically positioned within the tropical and subtropical zones of the NH, national influenza activity does not follow the typical temperate seasonal pattern. Surveillance data from WHO’s FluNet platform, which monitors influenza trends across 125 countries, indicate that influenza virus circulation in India occurs throughout the year, with detectable positivity rates during both the December-May and June-November periods [38].

To account for regional variation in influenza transmission, particularly in tropical and subtropical regions, the WHO’s Global Influenza Programme conducts technical consultations twice annually. These meetings, held in February and September, result in recommendations on the optimal influenza virus strains to be included in vaccines for the upcoming NH and SH influenza seasons, respectively.

For the tropics and subtropics, the most recent WHO influenza virus vaccine recommendation should be used for the administration of influenza vaccine, independent of the hemisphere in which the country is situated. This implies that if a patient approaches you approximately between September and February, the NH strain should be administered; and if a patient approaches you between March and August, the SH strain should be administered [39]. For this patient, the next influenza vaccine would be administered at the same time the following year. Since, on average, the antibodies to the influenza antigens in the vaccine, particularly for the H3 strain, last only about six months, and some locations experience year-round circulation of influenza, investigators are toying with the idea of twice-a-year vaccination to maintain higher levels of immunity and ward off infection [40].

What are the influenza vaccination recommendations in patients with COPD/asthma and other co-morbidities?

Influenza vaccination is highly recommended for patients with COPD, asthma, and other co-morbidities due to their increased susceptibility to severe influenza-related complications. The CDC advises annual influenza vaccination for all individuals aged 6 months and older, with particular emphasis on those with chronic respiratory conditions such as asthma and COPD [32,33]. The Ministry of Health and Family Welfare (MoHFW), Government of India, also emphasizes the critical role of influenza vaccination in individuals with chronic conditions such as asthma and COPD, as a preventive measure to reduce the risk of serious complications [41]. The WHO also advocates for annual influenza vaccination for high-risk groups, including those with chronic respiratory diseases. The Global Initiative for Chronic Obstructive Lung Disease (GOLD) recommends influenza vaccination for all COPD patients to reduce the risk of exacerbations and hospitalizations. Additionally, the Global Initiative for Asthma (GINA) highlights the necessity of influenza vaccination for asthma patients to prevent flu-related complications.

During the Joint Meeting of the Indian Chest Society and the National College of Chest Physicians held in January 2019, experts strongly advocated for the annual administration of a single dose of the quadrivalent influenza vaccine in adults. This recommendation is supported by evidence showing a 53% reduction in pneumonia, a 50% reduction in hospitalizations, and a 68% reduction in deaths from all causes during influenza outbreaks. Furthermore, the vaccine can prevent 59%-78% of asthma attacks that lead to emergency visits and/or hospitalizations [26]. Collectively, these organizations emphasize the critical role of influenza vaccination in protecting patients with chronic respiratory conditions and other co-morbidities.

What is the WHO’s stance on trivalent vs. quadrivalent inactivated influenza vaccines?

According to the most recent WHO guidance for the 2025-2026 NH influenza season, there has been a continued absence of confirmed detections of naturally circulating B/Yamagata lineage viruses since March 2020, indicating a minimal current risk of infection from this lineage [42]. Consistent with earlier recommendations, the WHO advisory group on influenza vaccine composition has concluded that the inclusion of the B/Yamagata antigen in quadrivalent influenza vaccines is no longer warranted and has advised its removal at the earliest feasible opportunity [43].

Similarly, updated guidance from the U.S. CDC confirms that, starting with the 2024-2025 influenza season, inactivated influenza vaccines distributed in the United States will no longer contain a B/Yamagata lineage virus or viral protein component. This decision is based on the absence of global surveillance detections of B/Yamagata viruses since early 2020 and the current assessment that the risk of human infection from this lineage remains extremely low [39,44].

Manufacturing of trivalent influenza vaccine will benefit the community at large in the following ways: (1) The persistent absence of confirmed detections of naturally occurring B/Yamagata lineage viruses mandates the inclusion of trivalent inactivated influenza vaccines in various national guidelines; (2) From the perspective of trivalent vaccines, a greater number of doses can be manufactured, which would improve immunization rates in India.

Is there a role for pharmacists, and how do they help in improving immunization? Is there any importance to having a dedicated vaccination room for adult vaccination?

In India, pharmacists play a pivotal role in enhancing immunization rates and promoting public health [45]. As easily accessible HCPs working under the supervision of registered medical practitioners, they contribute significantly to vaccine advocacy [46]. Pharmacists educate patients about the benefits and potential risks of vaccines, enabling informed decision-making and increasing the accessibility and convenience of immunization services [47]. Additionally, by organizing immunization clinics and collaborating with other healthcare providers, pharmacists help expand vaccine coverage and protect communities from vaccine-preventable diseases [48]. However, in India, pharmacists are bound by local laws and guidelines that prohibit them from administering any kind of injectables to patients, with or without the supervision of a registered medical practitioner.

The importance of a dedicated vaccination room for adult immunization is substantial in the Indian context [49]. Such rooms provide a private, hygienic, and comfortable environment for patients, which can help reduce anxiety and improve the overall vaccination experience [50]. With dedicated staff and proper infrastructure, these rooms ensure that vaccines are stored and administered under optimal conditions, thereby improving safety and efficiency [51]. This setup also helps reduce waiting times and fosters patient confidence, encouraging more adults to get vaccinated [52].

Establishing dedicated Adult Vaccination Clinics is essential for strengthening adult immunization efforts and improving public health outcomes in India [50]. These clinics facilitate streamlined and safe vaccination practices while fostering trust among patients through a professional and organized environment [53]. Pharmacists serve as integral members of the multidisciplinary healthcare team in these settings, working alongside doctors and nurses to advocate for immunization, educate the public, and administer vaccines under appropriate medical guidance [52]. Their involvement helps organize and manage immunization services efficiently, making them more accessible and increasing community participation [46]. This collaborative and structured approach significantly enhances vaccine coverage and protects the adult population from preventable diseases [54].

## Conclusions

The Congregation on Influenza Vaccination in Chronic Lung Diseases (CIRCLE) meet consensus emphasizes the critical importance of influenza vaccination, particularly for patients with chronic respiratory conditions such as COPD and asthma, as well as other co-morbidities. The document highlights the low vaccination rates in India and identifies significant challenges, including healthcare professionals' attitudes and public misconceptions about the efficacy and safety of influenza vaccines.

The consensus discusses the concept of year-long vaccination, noting that influenza viruses circulate year-round in India. The WHO recommends using the most recent inactivated influenza virus vaccine for administration, independent of the hemisphere. This implies administering the NH vaccine from September to February and the SH vaccine from March to August.

Guidelines from various health organizations, including the CDC, MoHFW, WHO, GOLD, and GINA, all advocate for annual influenza vaccination for high-risk groups to reduce the risk of severe complications, hospitalizations, and deaths. The document also highlights the significant reduction in pneumonia, hospitalizations, and deaths due to influenza vaccination, as well as the prevention of asthma attacks leading to emergency visits and hospitalizations.

The consensus from the CIRCLE meeting underscores the importance of a comprehensive strategy to enhance influenza vaccination coverage. Key recommendations include increasing public and professional awareness, strengthening education initiatives, improving healthcare delivery systems, and advancing supportive policy and advocacy measures. Given the continued absence of circulating B/Yamagata lineage viruses and the associated low risk of infection, the panel recommends prioritizing the use of trivalent influenza vaccines over quadrivalent formulations. Additionally, the meeting emphasizes the crucial role of pulmonologists across the country in addressing existing barriers to vaccination and in advancing efforts to improve national immunization rates and public health outcomes.
